# Anti-Melanoma Differentiation-Associated Gene 5 Dermatomyositis With Rapidly Progressive Interstitial Lung Disease Complicated by Pneumothorax, Pneumomediastinum, and Subcutaneous Emphysema: Clinical Course Compared With Reported Cases Worldwide

**DOI:** 10.7759/cureus.90433

**Published:** 2025-08-18

**Authors:** Reuben P Kumar, Vinay K Thallapally, Meghana Kesireddy, Sreekant Avula

**Affiliations:** 1 Primary Care, St. Francis Hospital, Muzaffarnagar, IND; 2 Rheumatology, HealthPartners Clinic, Saint Paul, USA; 3 Hematology and Medical Oncology, University of Nebraska Medical Center, Omaha, USA; 4 Internal Medicine, University of Minnesota, Minneapolis, USA

**Keywords:** anti-mda5 antibody, dermatomyositis, pneumomediastinum, pneumothorax, rapidly progressive interstitial lung disease, subcutaneous emphysema

## Abstract

Anti-melanoma differentiation-associated gene 5 (anti-MDA5) dermatomyositis (DM) represents a rare but clinically distinct subtype of idiopathic inflammatory myopathy. This condition is characterized by pathognomonic cutaneous manifestations, minimal muscle involvement, and an exceptionally high propensity for developing rapidly progressive interstitial lung disease (RP-ILD). We report a 49-year-old woman who presented with anti-MDA5 dermatomyositis complicated by RP-ILD and multiple severe thoracic complications, including recurrent pneumothorax, pneumomediastinum, and subcutaneous emphysema. Despite aggressive multimodal immunosuppressive therapy including high-dose corticosteroids, azathioprine, and mycophenolate mofetil (MMF), her clinical condition deteriorated rapidly. The patient required multiple interventions, including repeated chest tube placements, talc pleurodesis, and mechanical ventilation. Lung transplantation evaluation deemed her unsuitable for surgery, and she ultimately succumbed to hypoxic-hypercapnic respiratory failure within six months of initial presentation. This case underscores the aggressive natural history and guarded prognosis associated with anti-MDA5 dermatomyositis when complicated by RP-ILD and thoracic manifestations. The early recognition, prompt diagnosis, and immediate initiation of combination immunosuppressive therapy are paramount, although standardized treatment protocols remain elusive. Urgent research initiatives are needed to establish evidence-based therapeutic guidelines and improve patient outcomes in this challenging clinical entity.

## Introduction

Dermatomyositis (DM) represents a heterogeneous group of idiopathic inflammatory myopathies characterized by distinctive cutaneous lesions and variable systemic manifestations [1]. The clinical spectrum encompasses multiple subtypes, including classic dermatomyositis, cancer-associated dermatomyositis, clinically amyopathic dermatomyositis (CADM), and the increasingly recognized anti-melanoma differentiation-associated gene 5 (anti-MDA5) dermatomyositis [2].

Anti-MDA5 dermatomyositis constitutes a rare but clinically significant subtype of inflammatory myopathy, distinguished by its unique presentation of characteristic cutaneous manifestations, minimal or absent muscle involvement, and a disproportionately high risk of developing rapidly progressive interstitial lung disease (RP-ILD) [3,4]. This condition demonstrates notable resistance to conventional immunosuppressive therapies and carries a substantially elevated risk of life-threatening thoracic complications, including pneumomediastinum, pneumothorax, and subcutaneous emphysema.

Compared to other inflammatory myopathies, clinically amyopathic dermatomyositis associated with anti-MDA5 antibodies exhibits significantly higher morbidity and mortality rates [5]. The clinical course is often fulminant, with rapid progression to respiratory failure despite aggressive therapeutic interventions.

We present a comprehensive case report of a middle-aged woman with anti-MDA5 dermatomyositis complicated by RP-ILD and multiple concurrent thoracic complications, followed by a systematic review of similar cases in the literature to enhance the understanding of this challenging clinical entity.

## Case presentation

Initial presentation

A 49-year-old woman with a medical history significant for diabetes mellitus, asthma, and hypersensitivity lung disease presented to our emergency department in May 2022 with a six-month history of progressive symptoms. Her chief complaints included persistent fever, skin rashes, and worsening dyspnea.

Clinical history

The patient's symptom complex had evolved over the preceding six months, beginning with the insidious onset of a persistent rash on the face, neck, scalp, upper back, and hands accompanied by daily febrile episodes. Associated symptoms included pain, swelling and morning stiffness in multiple joints, unintentional weight loss of 65 pounds over six months, progressive dyspnea on exertion evolving to dyspnea at rest, persistent nonproductive cough, and dysphagia to both solids and liquids.

Physical examination

**Figure 1:**
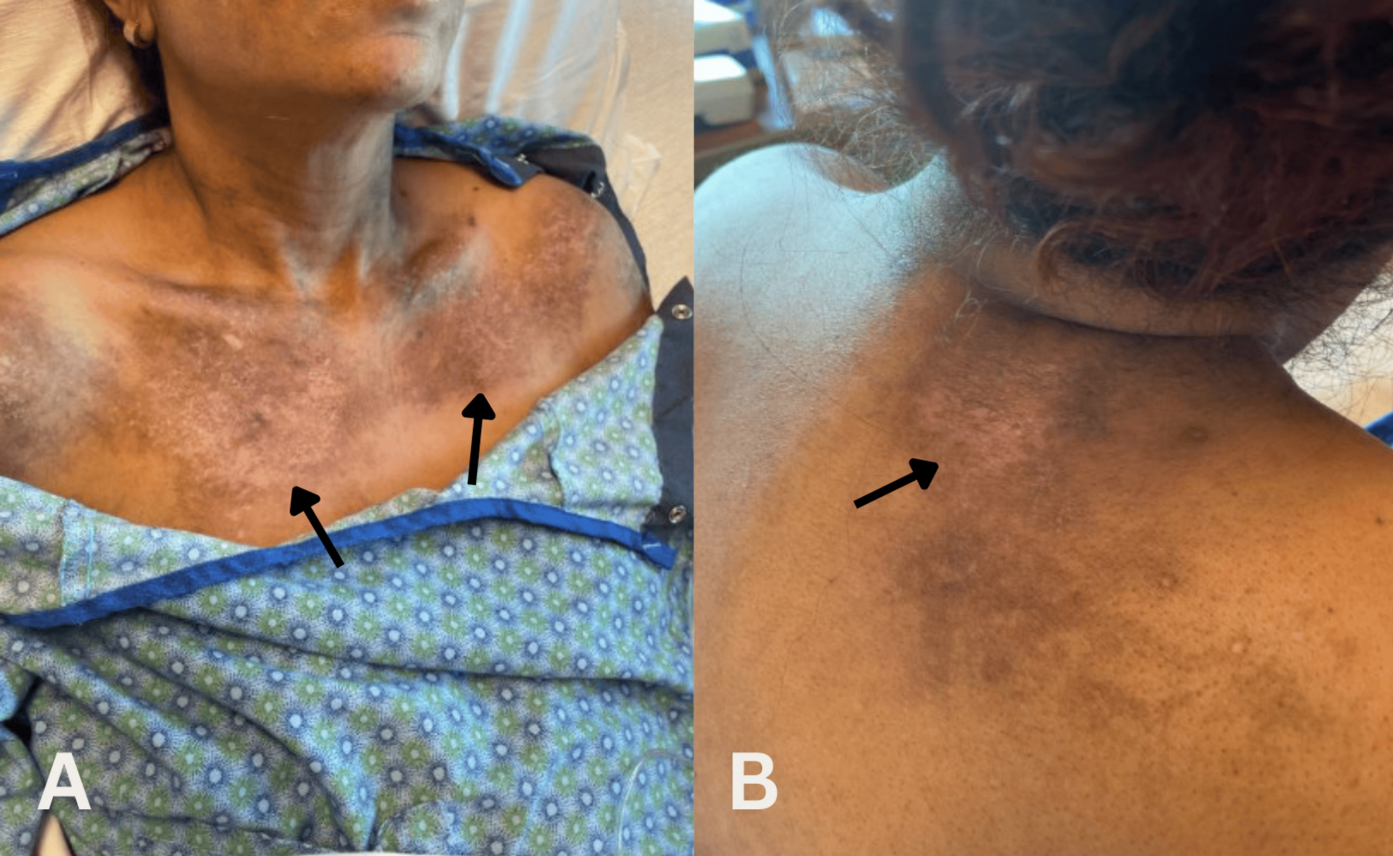
(A) Late shawl sign: hyperpigmented violaceous plaques over the neck and chest (black arrows). (B) Plaques over the upper back (black arrow)

**Figure 2:**
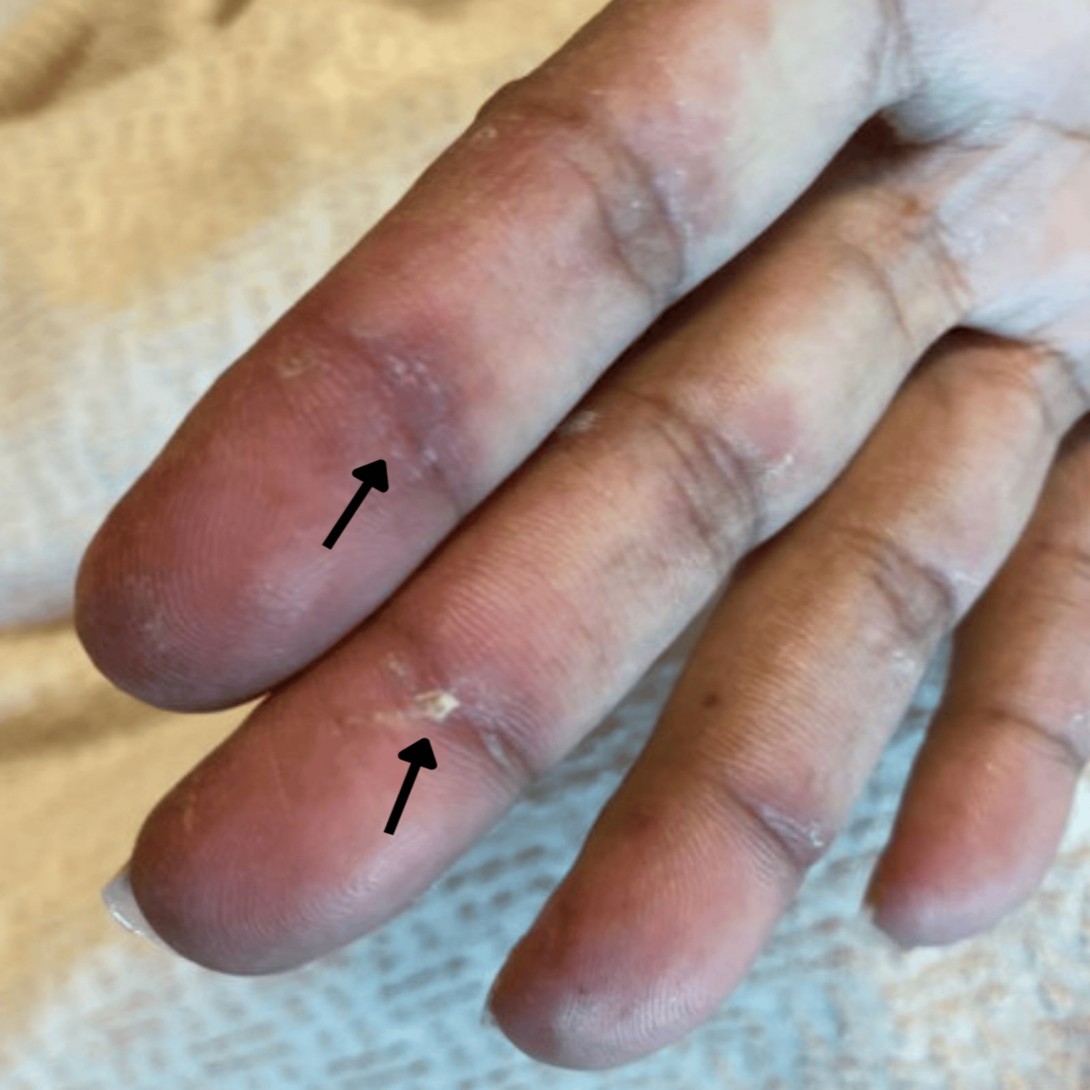
Mechanic's hands: rough, cracked, hyperpigmented skin over the palmar aspect of the fingers (black arrows)

Physical examination revealed distinctive dermatologic findings pathognomonic for dermatomyositis: hyperpigmented violaceous plaques distributed over the bilateral malar regions, neck, scalp, and upper back (Figure 1); characteristic "mechanic's hands" with rough, cracked, hyperpigmented skin over the palmar aspects of fingers (Figure 2); the absence of active Gottron's papules at presentation; and a requirement for 4 liters of supplemental oxygen to maintain adequate saturation.

Laboratory investigations

Initial laboratory studies revealed creatine kinase (CK) of 204 U/L (reference range: 29-168 U/L), inflammatory markers within normal limits (aldolase, 8.2 U/L; erythrocyte Sedimentation Rate, 2 mm/hour; C-reactive protein, 1.2 mg/L; and procalcitonin, 0.06 ng/mL), positive antinuclear antibody (ANA) with speckled pattern at 1:320 titer, positive Sjögren's syndrome antigen A (SSA), negative anti-double-stranded deoxyribonucleic acid (dsDNA), rheumatoid factor, anti-cyclic citrullinated peptide (CCP), antineutrophil cytoplasmic antibody (ANCA), hypersensitivity pneumonitis panel, and normal angiotensin converting enzyme (ACE) levels. Myositis-specific antibody testing showed a strongly positive anti-MDA5 antibody.

Imaging studies

**Figure 3:**
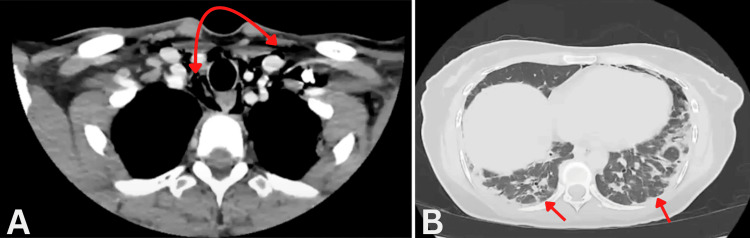
(A) Initial CT showing extensive subcutaneous emphysema throughout the visualized mediastinum and left pharyngeal space (red arrows). (B) Peripherally distributed bibasilar airspace opacities (red arrows) CT: computed tomography

Initial chest computed tomography (CT) demonstrated extensive subcutaneous emphysema involving the mediastinum and left pharyngeal space, with peripheral bibasilar airspace opacities consistent with early interstitial lung disease (Figure 3). Abdominal CT imaging excluded underlying malignancy. Magnetic resonance imaging of the thighs demonstrated marked edema in the proximal thigh muscles bilaterally.

Histopathologic findings

Skin biopsy revealed the lymphocytic infiltration of the dermis with increased mucin deposition, consistent with dermatomyositis.

Other findings

Bronchoalveolar lavage cultures were negative for infectious organisms.

Treatment course and clinical progression

Initial management included intravenous methylprednisolone 40 mg twice daily, transitioning to oral prednisone 60 mg daily, with azathioprine 50 mg once daily following the confirmation of normal thiopurine S-methyltransferase activity. The patient's clinical course was complicated by multiple serious thoracic manifestations: recurrent pneumothorax requiring chest tube insertion, multiple hospitalizations over subsequent weeks, and repeated chest tube placements with talc pleurodesis procedures.

**Figure 4:**
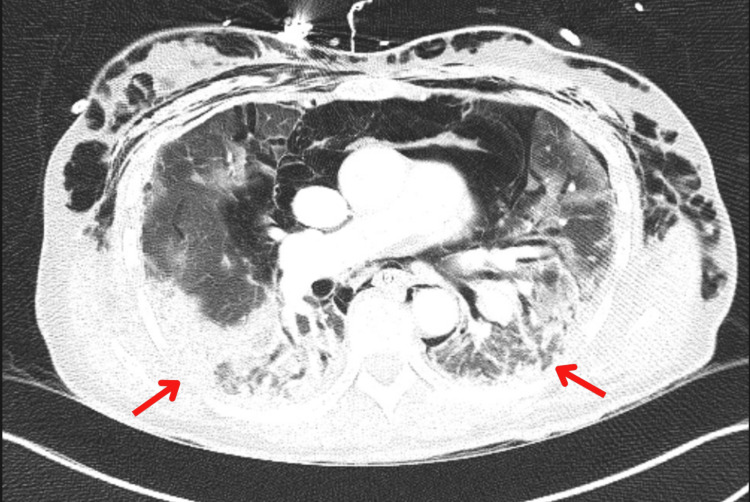
Repeat CT from a month later shows progressive interstitial lung disease (red arrows) CT: computed tomography

In June 2022, the patient was transferred to our tertiary care facility for escalated management. Repeat CT of the chest revealed progressive interstitial lung disease with increasing oxygen requirements (Figure 4). Treatment was modified to continue intravenous methylprednisolone 40 mg twice daily, discontinue azathioprine, and initiate mycophenolate mofetil (MMF) 1 g twice daily for two weeks, followed by 1.5 g twice daily. Empirical antibiotic therapy was administered, although the infectious workup remained negative.

Despite intensive interventions including surgical blowhole procedures and additional chest tube placements, the patient experienced progressive bilateral pneumothorax and progressive ILD. Escalation to high-flow nasal cannula and then bilevel positive airway pressure (BiPAP) support was required. Lung transplant evaluation deemed her unsuitable for surgery. The development of progressive encephalopathy and severe hypoxia necessitated intubation. Following multidisciplinary team discussions and family consultations, goals of care were transitioned to comfort-focused measures. The patient died within two months of hospitalization due to hypoxic-hypercapnic respiratory failure secondary to dermatomyositis-associated interstitial lung disease complicated by pneumomediastinum and bilateral pneumothorax.

## Discussion

Clinical significance of anti-MDA5 dermatomyositis

This case exemplifies the aggressive clinical phenotype associated with anti-MDA5 dermatomyositis, characterized by pathognomonic cutaneous manifestations. Anti-MDA5 antibodies demonstrate high specificity for dermatomyositis and correlate with a more aggressive disease course and significantly worse prognosis compared to other myositis subtypes.

Epidemiology and risk factors

Anti-MDA5 antibodies can be detected in 7%-60% of all dermatomyositis cases, with significant geographic and ethnic variation [5]. Anti-MDA5 dermatomyositis accounts for approximately 2% of all idiopathic inflammatory myopathies and demonstrates higher prevalence among Asian populations (particularly Japanese and Chinese) and women [3,6]. Critical epidemiologic findings include female predominance, geographic variation with higher prevalence in Asian populations, 20-fold higher odds of developing RP-ILD compared to MDA5-negative dermatomyositis [2], 50% of US patients with anti-MDA5 dermatomyositis being clinically amyopathic [7,8], and 20%-40% developing rapidly progressive interstitial lung disease.

Prognosis and mortality

Anti-MDA5 dermatomyositis with associated ILD carries an exceptionally poor prognosis, with six-month mortality rates ranging from 33% to 66%, primarily attributable to rapidly progressive respiratory failure despite aggressive immunosuppressive interventions [3].

Pathogenesis of thoracic complications

The pathophysiology underlying thoracic complications in dermatomyositis remains incompletely understood. Pneumomediastinum is more commonly associated with dermatomyositis than other connective tissue disorders [9], with the proposed mechanism being the rupture of subpleural cysts secondary to increased intra-alveolar pressure in the setting of preexisting alveolar damage [10]. Pneumothorax results from the rupture of pleural necrobiotic nodules or subpleural blebs, often recurring and proving refractory to conventional management [11]. Subcutaneous emphysema is reported in 55.5% of dermatomyositis cases [12], with anti-MDA5 antibody positivity representing a recognized risk factor [13].

Current treatment paradigms and future directions and evidence-based recommendations

Currently, no standardized treatment protocol exists for anti-MDA5 dermatomyositis. Recent evidence-based recommendations suggest first-line therapy with a triple combination of corticosteroids, calcineurin inhibitors, and intravenous immunoglobulin (IVIG), with alternative addition of intravenous cyclophosphamide as a fourth agent [14]. Second-line options include mycophenolate mofetil for calcineurin inhibitor intolerance and biologic agents such as rituximab (shown to reduce anti-MDA5 antibody titers) [15], and novel agents, including Janus kinase (JAK) inhibitors (tofacitinib) for refractory cases, show significant promise [16-19]. A systematic meta-analysis of seven studies on the use of JAK inhibitors in treating DM showed a significant decrease in skin manifestations and a significant increase in muscle strength. The response to tofacitinib further increases when used in combination with other immunosuppressants [20]. A random effect model data analysis was conducted on anti-MDA5 DM patients with ILD. Treatment with tofacitinib had a higher survival rate than control and showed improvement in lung function (forced vital capacity increased by 3.11%) [20]. JAK inhibitors showed clinical improvement in 94% of DM patients with ILD. Furthermore, anti-MDA5 DM patients on JAK inhibitors had a 100% six-month survival rate as compared to 78% in historical controls [21]. Rescue therapies encompass therapeutic plasma exchange, polymyxin B hemoperfusion, and combination immunosuppression protocols [22].

Challenges in management

Management challenges include resistance to therapy with 41% reported mortality despite treatment [23], the lack of standardization with no consensus treatment protocols, limited evidence from mostly case reports and small series, and supportive care limitations with no specific treatments for thoracic complications [24].

Literature review methodology

We conducted a comprehensive systematic search using multiple databases (Google Scholar, Medical Literature Analysis and Retrieval System Online {MEDLINE}, PubMed, and Scopus) spanning from January 2016 to January 2025. Search terms included combinations of the following: "Dermatomyositis", "Polymyositis", "Pneumothorax", "Pneumomediastinum", "Subcutaneous emphysema", "MDA5 dermatomyositis", "Interstitial lung disease", and "RP-ILD". No restrictions were applied regarding age, sex, geographic location, or language.

Results

Our literature review identified 25 case reports meeting the inclusion criteria (Table 1). Among the reviewed cases were the following: 13 (52%) men with a mean age of 46.4 years (standard deviation {SD}: ±14.9 years), a mean symptom duration of 35.3 months, interstitial lung disease present in 100% of cases, and rapidly progressive ILD in six cases (24%). Laboratory findings showed positive anti-Ro52 antibody in five cases (20%), elevated creatine kinase in 11 cases (44%), and normal CK in 11 cases (44%). Thoracic complications included pneumomediastinum in 22 cases (88%), subcutaneous emphysema in 14 cases (56%), pneumothorax in seven cases (28%), and isolated pneumomediastinum in six cases (24%). Clinical outcomes showed approximately 40% mortality despite aggressive interventions, with generally poor treatment response. Other parameters are further described in Table 2.

**Table 1:** Summary of cases of anti-MDA5 DM complicated with either pneumomediastinum, pneumothorax, or subcutaneous emphysema (sorted by year of publication) ↑CK levels of 500-1000 U/L ↑↑CK levels of 1001-2000 U/L ↑↑↑CK levels of >2000 U/L F, female; M, male; CK, creatine kinase; ILD, interstitial lung disease; RP-ILD, rapidly progressive ILD; Pm, pneumomediastinum; Pt, pneumothorax; SE, subcutaneous emphysema; GC, glucocorticoids; CyP, cyclophosphamide; Cyc A, cyclosporine A; MMF, mycophenolate mofetil; IVIG, intravenous immunoglobulin; ECMO, extracorporeal membrane oxygenation; AZA, azathioprine; N/A, not available

Serial number	Author	Year	Age (years)	Sex	Symptom duration (months)	Skin rash	CK levels	Ro52	ILD	RP-ILD	Pm	Pt	SE	Treatment	Outcome
1	Ma et al. [13]	2016	43	M	20	✓	↑↑↑	-	✓	-	✓	-	✓	GC, CyP, Cyc A, and MMF	Death
2	Ma et al. [13]	2016	42	M	3	✓	↑↑	-	✓	-	✓	-	-	GC, CyP, Cyc A, and MMF	Improved
3	Ma et al. [13]	2016	56	M	26	✓	↑	-	✓	-	✓	-	✓	GC, CyP, Cyc A, and MMF	Death
4	Ma et al. [13]	2016	38	F	48	✓	↑↑↑	-	✓	-	✓	-	-	GC, CyP, Cyc A, and MMF	Death
5	Ma et al. [13]	2016	74	M	3	✓	Normal	-	✓	-	✓	-	✓	GC, CyP, Cyc A, and MMF	Death
6	Ma et al. [13]	2016	44	M	7	✓	↑↑↑	-	✓	-	✓	-	✓	GC, CyP, Cyc A, and MMF	Death
7	Ma et al. [13]	2016	60	M	2	✓	Normal	-	✓	-	✓	-	-	GC, CyP, Cyc A, and MMF	Improved
8	Ma et al. [13]	2016	51	F	8	✓	↑↑↑	-	✓	-	✓	✓	✓	GC, CyP, Cyc A, and MMF	Death
9	Ma et al. [13]	2016	23	M	8	✓	Normal	-	✓	-	✓	-	✓	GC, CyP, Cyc A, and MMF	Improved
10	Ma et al. [13]	2016	42	F	10	✓	Normal	-	✓	-	✓	-	-	GC, CyP, Cyc A, and MMF	Improved
11	Sifuentes-Giraldo et al. [23]	2017	18	F	13	✓	Normal	✓	✓	-	✓	-	-	GC and AZA	Improved
12	Alqatari et al. [25]	2018	49	F	3	✓	↑	-	✓	-	✓	✓	-	GC and IVIG	Death
13	Farrell et al. [26]	2018	55	M	N/A	✓	N/A	-	✓	-	✓	✓	✓	N/A	N/A
14	Yashiro et al. [27]	2018	51	F	4	✓	↑	-	✓	✓	✓	-	-	GC, CyP, and Cyc A	Improved
15	Tandon et al. [28]	2019	41	F	4	✓	N/A	-	✓	-	-	✓	-	GC and IVIG	Improved
16	Fenando et al. [29]	2020	46	M	6	✓	Normal	-	✓	-	✓	-	✓	GC	Improved
17	Hwang et al. [30]	2020	48	M	4	✓	N/A	-	✓	-	✓	✓	✓	GC and MMF	Death
18	Saito et al. [31]	2021	67	F	6	✓	Normal	-	✓	✓	✓	✓	-	GC, CyP, and Cyc A	Death
19	Yeung et al. [32]	2021	16	F	2	✓	↑	✓	✓	-	✓	-	✓	GC, IVIG, Cyc A, and MMF	Improved
20	Al-Husayni et al. [33]	2022	46	M	4	✓	Normal	✓	✓	✓	-	✓	-	GC, CyP, and ECMO	Death
21	Kwon [34]	2022	42	M	1	✓	N/A	✓	✓	-	-	-	✓	GC and Cyc A	Improved
22	Rocha et al. [35]	2022	61	F	12	✓	Normal	-	✓	-	✓	-	✓	GC and MMF	Death
23	Tavakolian et al. [5]	2022	55	M	3	✓	↑	-	✓	✓	✓	✓	-	GC and MMF	Death
24	Mitra et al. [36]	2024	22	M	6	✓	Normal	✓	✓	✓	✓	✓	✓	-	Death
25	Subhadarshani et al. [37]	2025	71	F	N/A	✓	Normal	-	✓	✓	✓	-	✓	GC and AZA	Improved
26	Our case	2022	49	F	6	✓	Normal	-	✓	✓	✓	✓	✓	GC, AZA, and MMF	Death

**Table 2:** Characteristics of cases described in the review of literature -: no relevant details available SD, standard deviation; N, number; CK, creatine kinase; ILD, interstitial lung disease

Parameters	N (%)	Details
Demographics	-	-
Mean age + SD	44.0 + 11.0 years	Range: 17-62 years
Female sex	13 (52.0%)	-
Male sex	12 (48.0%)	-
Clinical features	-	-
Pneumomediastinum	22 (88.0%)	Most common thoracic complication
Subcutaneous emphysema	16 (64.0%)	Second most common
Pneumothorax	7 (28.0%)	Often recurrent when present
Rapidly progressive ILD	7 (28.0%)	Associated with poor prognosis
Laboratory findings	-	-
Elevated CK	11 (44.0%)	-
Normal CK	11 (44.0%)	-
Positive Ro52	5 (20.0%)	Among the tested cases
Outcomes	-	-
Death	10 (40.0%)	Most within six months
Clinical improvement	8 (32.0%)	Variable response to treatment
Stable disease	7 (28.0%)	-
Mean follow-up	15.3 months	Range: 3-25 months

Future research priorities

Future research priorities include large-scale clinical trials to establish optimal therapeutic regimens, biomarker development for early detection and monitoring, personalized medicine approaches based on genetic and immunologic profiles, novel therapeutic targets including pathway-specific interventions, and quality of life studies with long-term outcome assessments.

## Conclusions

This case report and comprehensive literature review highlight several critical clinical insights: early recognition is paramount, as characteristic cutaneous findings combined with rapid respiratory deterioration should prompt immediate evaluation for anti-MDA5 antibodies. Aggressive intervention is required, with combination immunosuppressive therapy initiated promptly, though optimal regimens remain undefined. Prognosis remains guarded, as despite intensive interventions, mortality rates are high in this patient population. Research gaps persist, with standardized treatment protocols urgently needed based on high-quality evidence. The complexity and poor outcomes of anti-MDA5 dermatomyositis highlight the need for multicenter research to develop evidence-based treatments. Future studies should aim to develop new and optimal treatment strategies to improve patient outcomes. Healthcare providers must be vigilant for this condition in patients with characteristic skin and respiratory symptoms, as early intervention may be the only opportunity to alter the disease course.

## References

[1] DeWane ME, Waldman R, Lu J (2020). Dermatomyositis: clinical features and pathogenesis. J Am Acad Dermatol.

[2] Mehta P, Machado PM, Gupta L (2021). Understanding and managing anti-MDA 5 dermatomyositis, including potential COVID-19 mimicry. Rheumatol Int.

[3] Wu W, Guo L, Fu Y (2021). Interstitial lung disease in anti-MDA5 positive dermatomyositis. Clin Rev Allergy Immunol.

[4] McPherson M, Economidou S, Liampas A, Zis P, Parperis K (2022). Management of MDA-5 antibody positive clinically amyopathic dermatomyositis associated interstitial lung disease: a systematic review. Semin Arthritis Rheum.

[5] Tavakolian K, Odak M, Mararenko A, Ilagan J, Douedi S, Khan T, Al Saoudi G (2022). Anti-MDA5 associated clinically amyopathic dermatomyositis with rapidly progressive interstitial lung disease. J Med Cases.

[6] Tiniakou E, Mecoli CA, Kelly W (2023). Anti-MDA5-positive dermatomyositis and remission in a single referral centre population. Clin Exp Rheumatol.

[7] Fiorentino D, Chung L, Zwerner J, Rosen A, Casciola-Rosen L (2011). The mucocutaneous and systemic phenotype of dermatomyositis patients with antibodies to MDA5 (CADM-140): a retrospective study. J Am Acad Dermatol.

[8] Moghadam-Kia S, Oddis CV, Sato S, Kuwana M, Aggarwal R (2017). Antimelanoma differentiation-associated gene 5 antibody: expanding the clinical spectrum in North American patients with dermatomyositis. J Rheumatol.

[9] Okamoto S, Tsuboi H, Noma H (2021). Predictive factors for pneumomediastinum during management of connective tissue disease-related interstitial lung disease: a retrospective study. Intern Med.

[10] Lee SZ, Syed MT, Kumar P (2021). Pneumomediastinum: a severe complication of dermatomyositis. Int J Case Rep Images.

[11] Bradley JD (1986). Spontaneous pneumomediastinum in adult dermatomyositis. Ann Rheum Dis.

[12] Subki AH, Almani IM, Albeity A, Aljabri BK, Alsolaimani R, Halabi H (2023). Spontaneous pneumomediastinum and subcutaneous emphysema in dermatomyositis: a case series and literature review. J Inflamm Res.

[13] Ma X, Chen Z, Hu W, Guo Z, Wang Y, Kuwana M, Sun L (2016). Clinical and serological features of patients with dermatomyositis complicated by spontaneous pneumomediastinum. Clin Rheumatol.

[14] Romero-Bueno F, Diaz Del Campo P, Trallero-Araguás E (2020). Recommendations for the treatment of anti-melanoma differentiation-associated gene 5-positive dermatomyositis-associated rapidly progressive interstitial lung disease. Semin Arthritis Rheum.

[15] He C, Li W, Xie Q, Yin G (2021). Rituximab in the treatment of interstitial lung diseases related to anti-melanoma differentiation-associated gene 5 dermatomyositis: a systematic review. Front Immunol.

[16] Selva-O'Callaghan A, Romero-Bueno F, Trallero-Araguás E, Gil-Vila A, Ruiz-Rodríguez JC, Sánchez-Pernaute O, Pinal-Fernández I (2021). Pharmacologic treatment of anti-MDA5 rapidly progressive interstitial lung disease. Curr Treatm Opt Rheumatol.

[17] Sabbagh S, Almeida de Jesus A, Hwang S (2019). Treatment of anti-MDA5 autoantibody-positive juvenile dermatomyositis using tofacitinib. Brain.

[18] Nombel A, Fabien N, Coutant F (2021). Dermatomyositis with anti-MDA5 antibodies: bioclinical features, pathogenesis and emerging therapies. Front Immunol.

[19] Corbella-Bagot L, Bosch-Amate X, Gimeno-Ribes E (2024). JAK inhibitors in refractory dermatomyositis: a case series. Australas J Dermatol.

[20] Ma C, Liu M, Cheng Y, Wang X, Zhao Y, Wang K, Wang W (2024). Therapeutic efficacy and safety of JAK inhibitors in treating polymyositis/dermatomyositis: a single-arm systemic meta-analysis. Front Immunol.

[21] Paik JJ, Lubin G, Gromatzky A, Mudd PN Jr, Ponda MP, Christopher-Stine L (2023). Use of Janus kinase inhibitors in dermatomyositis: a systematic literature review. Clin Exp Rheumatol.

[22] Shirakashi M, Nakashima R, Tsuji H (2020). Efficacy of plasma exchange in anti-MDA5-positive dermatomyositis with interstitial lung disease under combined immunosuppressive treatment. Rheumatology (Oxford).

[23] Sifuentes-Giraldo WA, García-Villanueva MJ, Gorospe L (2017). Spontaneous pneumomediastinum complicating interstitial lung disease, associated with clinically amyopathic dermatomyositis and positive anti-MDA5 antibodies. Rev Colomb Reumatol.

[24] Pallo PA, Shinjo SK (2018). Spontaneous pneumomediastinum in dermatomyositis: a case series and literature review. Med Express.

[25] Alqatari S, Riddell P, Harney S, Henry M, Murphy G (2018). MDA-5 associated rapidly progressive interstitial lung disease with recurrent pneumothoraces: a case report. BMC Pulm Med.

[26] Farrell S, Huprikar N, Hiles P (2018). MDA-5 positive dermatomyositis complicated by pneumomediastinum. Am J Respir Crit Care Med.

[27] Yashiro M, Asano T, Sato S, Kobayashi H, Watanabe H, Miyata M, Migita K (2018). Anti-MDA5 antibody-positive hypomyopathic dermatomyositis complicated with pneumomediastinum. Fukushima J Med Sci.

[28] Tandon VO, Nadler E, Tandon A, Manek G, Grover P (2019). Rapidly progressive interstitial lung disease associated with amyopathic dermatomyositis. Am J Respir Crit Care Med.

[29] Fenando A, Firn K, Louissa S, Hussain A (2020). Case of anti-MDA-5 positive dermatomyositis with rapidly progressive interstitial lung disease. BMJ Case Rep.

[30] Hwang J, Achamallah N (2020). Holy Macklin! Severe pneumomediastinum associated with anti-MDA-5 antibody dermatomyositis. Chest.

[31] Saito T, Mizobuchi M, Miwa Y (2021). Anti-MDA-5 antibody-positive clinically amyopathic dermatomyositis with rapidly progressive interstitial lung disease treated with therapeutic plasma exchange: a case series. J Clin Apher.

[32] Yeung TW, Cheong KN, Lau YL, Tse KN (2021). Adolescent-onset anti-MDA5 antibody-positive juvenile dermatomyositis with rapidly progressive interstitial lung disease and spontaneous pneumomediastinum: a case report and literature review. Pediatr Rheumatol Online J.

[33] Al-Husayni F, Munshi A, Qanash S, Shaikhain TA, Alzahrani Z, Alghamdi B (2022). Clinically amyopathic dermatomyositis with rapid progressive interstitial lung disease diagnosed in a patient on extracorporeal membrane oxygenation. Cureus.

[34] Kwon M (2022). Spontaneous pneumomediastinum and cutaneous ulcers complicated in a patient with dermatomyositis and interstitial lung disease. Arch Rheumatol.

[35] Rocha ML, Gago L, Sepriano A (2022). Spontaneous pneumomediastinum, a rare manifestation of clinically amyopathic dermatomyositis. ARP Rheumatol.

[36] Mitra S, Parvathy N, Garg M, Devkota S, Bansal S, Sehgal IS, Gupta K (2024). Anti MDA-5 associated rapidly progressive interstitial lung disease complicated by viral pneumonia - a fatal outcome. Autops Case Rep.

[37] Subhadarshani S, Woodie B, Bookal E, Reed J (2025). Subcutaneous emphysema and severe interstitial lung disease in the setting of anti-MDA 5 positive dermatomyositis in a Hispanic patient. Case Rep Rheumatol.

